# A Practical Treatise on the Diseases of the Testis and of the Spermatic Cord and Scrotum, with Numerous Wood Engravings

**Published:** 1856-10

**Authors:** 


*A Practical Treatise on the Diseases of the Testis and of the Spermatic Cord and Scrotum, with numerous wood engravings.* By F. B. Curling, F.R.S., Surgeon to the London Hospital, &c., &c. Second American from the second revised and enlarged English Edition. Philadelphia, Blanchard & Lea, 1856.

It is unnecessary for us to say anything in favor of this truly excellent work of Mr. Curling's. The American profession are already acquainted with its merits, and the simple announcement of a new edition is sufficient to secure for it that attention which its merits deserve.

For sale by KEEN & LEE. J.

